# The predictive value of heparin-binding protein for bacterial infections in patients with severe polytrauma

**DOI:** 10.1371/journal.pone.0300692

**Published:** 2024-12-26

**Authors:** Li Li, Xiao-xi Tian, Gui-long Feng, Bing Chen

**Affiliations:** 1 Department of Critical Care Medicine, The Second Hospital of Tianjin Medical University, Tianjin, China; 2 Department of Emergency, The First Hospital of Shanxi Medical University, Taiyuan, Shanxi, China; Pescara General Hospital, ITALY

## Abstract

**Introduction:**

Heparin-binding protein is an inflammatory factor with predictive value for sepsis and participates in the inflammatory response through antibacterial effects, chemotaxis, and increased vascular permeability. The role of heparin-binding protein in sepsis has been progressively demonstrated, but few studies have been conducted in the context of polytrauma combined with bacterial infections. This study aims to investigate the predictive value of heparin-binding protein for bacterial infections in patients with severe polytrauma.

**Materials and methods:**

This is a prospective single-center study. Patients with polytrauma in the emergency intensive care unit were selected for the study, and plasma heparin-binding protein concentrations and other laboratory parameters were measured within 48 hours of admission to the hospital. A two-sample comparison and univariate logistic regression analysis investigated the relationship between heparin-binding protein and bacterial infection in polytrauma patients. A multifactor logistic regression model was constructed, and the ROC curve was plotted.

**Results:**

Ninety-seven patients with polytrauma were included in the study, 43 with bacterial infection and 54 without infection. Heparin-binding protein was higher in the infected group than in the control group [(32.00±3.20) ng/mL vs. (18.52±1.33) ng/mL, P = 0.001]. Univariate logistic regression analysis shows that heparin-binding protein is related to bacterial infection (OR = 1.10, Z = 3.91, 95%CI:1.05~1.15, P = 0.001). Multivariate logistic regression equations showed that patients were 1.12 times more likely to have bacterial infections for each value of heparin-binding protein increase, holding neutrophils and Procalcitonin (PCT) constant. ROC analysis shows that heparin-binding protein combined with neutrophils and PCT has better predictive value for bacterial infection [AUC = 0.935, 95%CI:0.870~0.977].

**Conclusions:**

Heparin-binding protein may predict bacterial infection in patients with severe polytrauma. Combining heparin-binding protein, PCT, and neutrophils may improve bacterial infection prediction.

## Introduction

Polytrauma is the fourth leading cause of death, accounting for 11.2% of disability-adjusted life-years worldwide [1, 2]. Infection and inflammatory response after polytrauma greatly impact the prognosis. Therefore, monitoring inflammatory indicators can help early identification of infection and timely adjustment of treatment strategies [3]. Although the Advanced Trauma Life Support clinical guideline and the Injury Severity Score can assess the condition and guide interventions, more reliable indicators of infection are needed for early prediction of infection [4]. Given the critical role of inflammatory factors in multiple injuries [5], using reliable indicators to predict infection is essential for early clinical intervention and reduction of mortality.

Heparin-binding protein (HBP) is mainly produced and stored in neutrophils [6]. It is released in response to numerous cytokines, inflammatory agents, chemokines, and microorganisms. Monocytes can also release small amounts of it [7, 8]. HBP can chemotaxis neutrophil migration to exert antimicrobial effects [6]. In addition, HBP enhances the release of pro-inflammatory factors mediated by NF-KB in M1 macrophages [9]. Previous studies have found that HBP correlates with the severity of sepsis, community-acquired pneumonia, bacterial meningitis, and COVID-19 [10]. Common markers of infection in the clinical setting, such as C-reactive protein (CRP) and procalcitonin (PCT), have good negative predictive accuracy within 36 hours of infection [11]. Other markers, including white blood cell (WBC) levels, interleukin (IL)-6, IL-8, and serum amyloid (SAA), are also indicative of post-infectious inflammation to varying degrees [12]. Studies have shown that the combination of HBP with CRP and PCT can predict severe acute pancreatitis [13]. The diagnostic and prognostic predictive role of heparin-binding protein in sepsis has been progressively demonstrated, but few studies have been conducted in the context of polytrauma combined with bacterial infections [14, 15].

The purpose of this study was to determine whether HBP predicts bacterial infection in patients with severe polytrauma. For this purpose, we prospectively studied the correlation between HBP and bacterial infection and selected variables with clinical value to construct a logistic regression model. The calibration curve and ROC curve are used to evaluate the accuracy and predictive value of the model, respectively. It provides a reference value for predicting bacterial infections in emergency polytrauma in clinical practice.

## Methods

### Sample sources and ethical issues

This is a prospective study of patients with polytrauma directly admitted to the Emergency Intensive Care Unit (EICU) of the First Hospital of Shanxi Medical University from November 2022 to October 2023. The average number of patients admitted to the EICU is about 432 annually, with 48 cases per month, from which the research criteria were screened. All patients were treated in a standardized manner by a multidisciplinary team and all received ventilator-assisted respiration. Case screening was performed simultaneously by three doctors, two performed data entry and proofreading with data from the nursing department’s custody records. Polytrauma is defined as trauma to two or more anatomical parts or organs of the body under the action of the same causative agent, where at least one of the injuries can be life-threatening. This study focused on critically ill polytrauma patients admitted to the EICU because of their critical condition. All procedures were performed following relevant laws and institutional guidelines, in compliance with the World Medical Association Declaration of Helsinki, and were approved by the Ethics Committee of the First Hospital of Shanxi Medical University [2021 Lun Review Words (K-K115)]. Case details and personal information included in this study were obtained with consent and authorization. Written consent for this study was obtained from the participants involved or the patients’ families.

The outcome indicators were the patients’ blood, cerebrospinal fluid, and sputum culture findings. Patients with no positive microbiologic culture findings were placed in the control group, while those with positive bacteria culture results were placed in the infection group.

### Inclusion and exclusion criteria

The inclusion criteria included the following: Age ≥18 years; time from injury to admission <24 hours; patients diagnosed with severe polytrauma; first hospitalization for polytrauma. Exclusion Criteria: Patients with a hospital stay of less than three days or who are certified dead upon resuscitation; patients transferred from outside hospitals for treatment; patients with other infections that are not bacterial; prior use of anticoagulant medications; no antibiotic treatment before admission; infectious disease before the current trauma; history of severe trauma or surgery in the last three months; diseases that affect coagulation or immunity.

### Sample detection and data collection

All samples were collected within 48 hours of admission, specifically at 6 AM Beijing time on the first or second day of hospitalization, either through peripheral venipuncture or before surgery. A 5 ml sodium citrate tube was used for blood collection, avoiding heparin-containing indwelling needles. Samples were centrifuged within 5 minutes of blood collection (5200 r/min, 5 min). After centrifugation, the plasma was taken 20μl and refrigerated at 2~8°C for five days. The HBP assay was carried out in batches using rate-scattering turbidimetric assay to reduce the error. The EZ-400 Semi-Automatic Specific Protein Analyzer has 4 reaction cups for simultaneous testing. Two levels of QC are tested simultaneously after swipe calibration, and the results obtained have a relative deviation of ≤10% before the sample can be tested. The sample is divided into two portions with a minimum dosage of 5 μL each and assayed simultaneously in adjacent channels. The Heparin Binding Protein Assay Kit is given a linear interval that the lower limit should be no more than 6 ng/mL, the upper limit should be no less than 300 ng/mL), the correlation coefficient (r) should be no less than 0.9900, and the coefficient of variation (CV) ≤10% (The reagents and instruments used to detect HBP in this study were obtained from Suzhou Kangheshun Medical Co.). Patient clinical information is based on information available during this admission. Laboratory indicators are based on the return of laboratory results within 48 hours of our hospital.

### Statistical analysis

Stata MP 16 was used to carry out all statistical evaluations and visualizations. Quantitative was described as mean ± standard deviation after assessing the normality of the quantitative data, and categorical data was described using counts and percentages. Quantitative comparisons were made using the two independent samples t-test or t-test. Qualitative comparisons were made using the χ2 or Fisher’s exact test. P<0.05 was considered statistically significant.

Univariate logistic analyses were performed on variables whose differences were statistically significant after comparing the two samples. The multivariate logistic regression model was constructed using forward stepwise regression to screen the significant variables after univariate logistic regression analysis. Hosmer-Lemeshow test was used to test the goodness of fit of the model. The calibration curve and ROC curve were used to visualize and evaluate the predictive efficacy of the logistic regression model.

## Result

### Clinical characteristics and comparison of two groups

After screening and testing, 97 polytrauma patients were eligible and participated in this study. The median time from the onset of trauma to report to the hospital was (71.73±4.34) minutes. Microbiological cultures were performed based on the patient’s blood, sputum, and cerebrospinal fluid, and 43 were eventually included in the bacterial infection group and 54 in the control group. The clinical characteristics and comparison of the two groups are shown in Table 1. HBP was higher in the infected group than in the control group, and the difference was statistically significant [(32.00±3.20) ng/mL vs. (18.52±1.33) ng/mL, P = 0.001]. Age, smoking, respiratory rate, HBP, leukocytes, neutrophils, and Procalcitonin (PCT) were also significantly higher in the infected group (p<0.05). The control group had a higher Glasgow Coma Scale (GCS) score than the infected group, and the difference was statistically significant(P<0.05). Gender, BMI, temperature, pulse, Injury Severity Score (ISS) score, alcohol consumption, hypertension, diabetes mellitus, lymphocytes, calcium, erythrocytes, hemoglobin, platelets, Prothrombin Time (PT), Prothrombin Time—International Normalized Ratio (PTINR), Activated Partial Thromboplastin Time (APTT), Thrombin Time (TT), Fibrin Degradation Products (FDP), D-Dimer (DD), and Fibrinogen (FIB) had no statistically significant difference between the two groups(P>0.05).

**Table 1:** Clinical characteristics and comparison of two groups.

	Infection group	Control group	Overall	*P*
	(*n* = 43)	(*n* = 54)	(*n* = 97)	
**Gender, *n (%)***				0.346
Female	7 (16.28)	13 (24.07)	20 (20.62)	
Male	36 (83.72)	41 (75.93)	77 (79.38)	
**Age, years**	58.88±2.40	51.80±1.98	54.94±1.56	**0.008**
**BMI (kg/m** ^**2**^ **)**	23.44±0.39	24.49±0.49	24.02±0.33	0.095
**Body temperature (°C)**	36.67±0.08	36.60±0.07	36.63±0.05	0.854
**Pulse (bpm)**	83.12±2.94	81.76±2.12	82.36±1.75	0.931
**Respiration (bpm)**	19.65±0.63	18.15±0.56	18.81±0.42	**0.019**
**ISS**	20.42±1.19	18.28±1.17	19.23±0.84	0.094
**GCS**	8.72±0.57	11.20±0.41	10.10±0.16	**0.001**
**Tobacco Smoking, *n (%)***	27 (62.79)	20 (37.04)	47 (48.45)	**0.012**
**Alcohol Drinking, *n (%)***	18 (41.86)	13 (24.07)	31 (31.96)	0.062
**Comorbidities, *n (%)***				
Hypertension	24 (55.81)	22 (40.74)	46 (47.42)	0.140
Diabetes	8 (18.60)	5 (9.26)	13 (13.40)	0.180
**Peripheral blood**				
HBP (ng/mL)	32.00±3.20	18.52±1.33	24.5±1.73	**0.001**
WBC (10^9^/L)	14.49±0.79	8.96±0.50	11.41±0.53	**0.001**
Lymphocyte (10^9^/L)	1.41±0.39	1.45±0.23	1.43±0.21	0.156
Neutrophil (10^9^/L)	12.36±0.75	6.84±0.51	9.29±0.52	**0.001**
PCT (10^9^/L)	1.10±0.17	0.27±0.04	0.64±0.09	**0.001**
Ca (mg/dL)	2.20±0.02	2.23±0.02	2.22±0.01	0.383
RBC (10^12^/L)	4.07±0.21	4.12±0.11	4.1±0.11	0.287
HB (g/L)	121.20±3.43	123.32±3.39	122.38±2.41	0.703
PLT (10^9^/L)	197.67±14.86	193.35±7.80	195.26±7.85	0.470
PT (s)	14.80±0.43	14.65±0.17	14.72±0.21	0.850
PTINR	1.19±0.10	1.14±0.08	1.16±0.06	0.569
APTT (s)	27.24±0.57	26.76±0.38	26.97±0.33	0.700
TT (s)	15.51±0.34	15.55±0.31	15.53±0.23	0.862
FDP (ug/ml)	27.73±3.55	21.70±2.34	24.37±2.05	0.309
DD (ng/ml)	2113.28±311.89	1805.07±239.24	1941.70±191.58	0.452
FIB c (g/L)	3.40±0.17	3.10±0.10	3.23±0.10	0.110
**Surgery, *n (%)***	31 (72.09)	29 (53.70)	60 (61.86)	0.064

Data are presented as numbers, percentages, and mean ± standard deviation; ISS, Injury Severity Score; GCS, Glasgow Coma Scale; HBP, heparin-binding protein; WBC, white blood cell; PCT, procalcitonin; Ca, Calcium; RBC, red blood cell; HB, hemoglobin; PLT, blood platelet; PT, prothrombin time; PTINR, prothrombin time and international normalized ratio; APTT, activated partial thromboplastin time; TT, thrombin time; FDP, fibrinogen degradation product; DD, D dimer; FIBc, fibrinogen.

A total of 43 patients were identified in the bacterial infection group by patient blood, sputum, and cerebrospinal fluid cultures. Bacterial species and distribution are shown in Table 2. Among the patients with positive bacterial cultures, eight patients had multiple bacterial infections. More patients had positive sputum cultures for bacteria (n = 39) than cerebrospinal fluid cultures (n = 8), and fewer patients had positive blood cultures for bacteria (n = 4). In addition, the most common result for sputum cultures that were positive for bacteria was Klebsiella pneumoniae (n = 16), and the most common bacteria for cerebrospinal fluid infections was Staphylococcus (n = 5). Bacteria positive in blood culture include Staphylococcus, Enterobacteriaceae, Fungal, and Bacillus.

**Table 2:** Bacterial species and distribution.

Bacterial species	Sputum culture	Blood Culture	Cerebrospinal Fluid
Staphylococcus (*n*)	4	1	5
Klebsiella pneumoniae (*n*)	16	0	1
Corynebacterium striatum (*n*)	5	0	0
Moraxellaceae (*n*)	4	0	0
Acinetobacter baumannii (*n*)	2	0	1
Corynebacterium striatum (*n*)	2	0	0
Enterobacter cloacae (*n*)	1	0	0
Acinetobacter lwoffii (*n*)	1	0	1
Escherichia coli (*n*)	1	1	1
Stenotrophomonas (*n*)	1	0	0
Fungal (*n*)	1	1	1
Haemophilus (*n*)	1	0	0
Bacillus (*n*)	0	1	0
Count (*n*)	39	4	10

### Univariate logistic regression analysis

Univariate logistic regression was used to analyze the variables with statistically significant differences between the infection and control groups, as shown in Table 3. The results show that age, GCS score, smoking, HBP, WBC, neutrophils, and PCT are related to bacterial infection in polytrauma (all P<0.05). However, Respiration has no significant correlation with polytrauma infection (OR = 1.10, Z = 1.70, 95%CI:0.99~1.22, P = 0.09). Furthermore, for every unit increase of HBP, there was a 10% increase in the risk of bacterial infection (OR = 1.10, Z = 3.91, 95%CI:1.05~1.15, P = 0.001). This confirms that the HBP difference between the infected and control groups was statistically significant.

**Table 3:** Univariate logistic regression analysis.

	*Odds ratio*	*Standard Error*	*Z*	*P>Z*	[*95% conf*. *interval*]	
Age, years	1.03	0.02	2.19	0.028	1.00	1.06
Respiration (bpm)	1.10	0.06	1.70	0.090	0.99	1.22
GCS	0.81	0.05	-3.27	0.001	0.71	0.92
Tobacco Smoking, *n* (*%*)	2.87	1.21	2.49	0.013	1.25	6.57
HBP (ng/mL)	1.10	0.03	3.91	0.001	1.05	1.15
WBC (10^9^/L)	1.44	1.12	4.34	0.001	1.22	1.70
Neutrophil (10^9^/L)	1.44	0.12	4.41	0.001	1.22	1.69
PCT (10^9^/L)	24.47	17.92	4.37	0.001	5.83	102.78

GCS, Glasgow Coma Scale; HBP, heparin-binding protein; WBC, white blood cell; PCT, procalcitonin.

### Multivariable logistic regression model

In order to further screen out variables with statistical significance and clinical value, the variables after univariate logistic analysis were screened using forward stepwise regression, and a multivariate logistic regression model was obtained (Table 4). The variables screened out by forward stepwise regression included HBP, neutrophils, and PCT. Multivariate logistic regression equations showed that patients were 1.12 times more likely to have bacterial infections for each value of heparin-binding protein increase, holding neutrophils and PCT constant.

**Table 4:** Multivariable logistic regression model.

	*Odds ratio*	*Standard Error*	*Z*	*P*	[*95% conf*. *interval*]	
**HBP** (ng/mL)	1.118	0.042	2.95	0.003	1.038	1.205
**Neutrophil** (10^9^/L)	1.204	0.090	2.47	0.013	1.039	1.395
**PCT** (10^9^/L)	53.876	57.207	3.75	0.001	6.723	431.735
**constant**	0.001	0.002	-4.69	0.001	0.001	0.021

HBP, heparin-binding protein; PCT, procalcitonin.

### Hosmer-Lemeshow test and calibration curve

**Fig 1:**
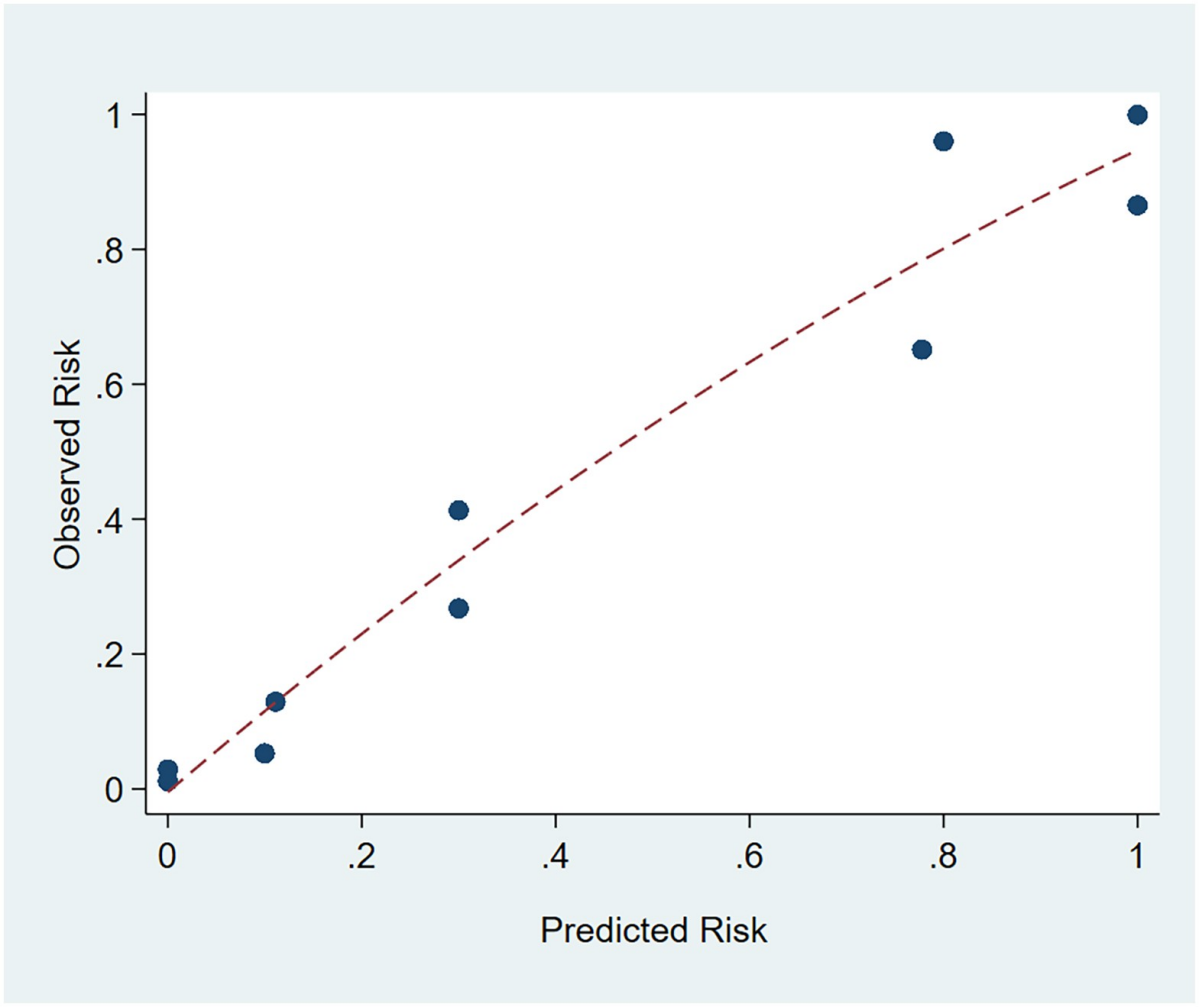
Calibration curve of multivariable logistic regression model.

The Hosmer-Lemeshow test (HL test) was used to analyze the goodness of fit of the multivariable logistic regression model. The results show that the P value is insignificant, so the PH assumption cannot be violated (χ2 = 86.96, P = 0.6568>0.05). The model passes the HL test, and the model fit is good. The calibration curve is plotted to visualize the logistic regression model based on the results of the HL goodness-of-fit test (Fig 1). The deviation between predicted and observed risk is slight and close to the reference line, indicating that the model’s predictive ability is good.

### ROC analysis

**Fig 2:**
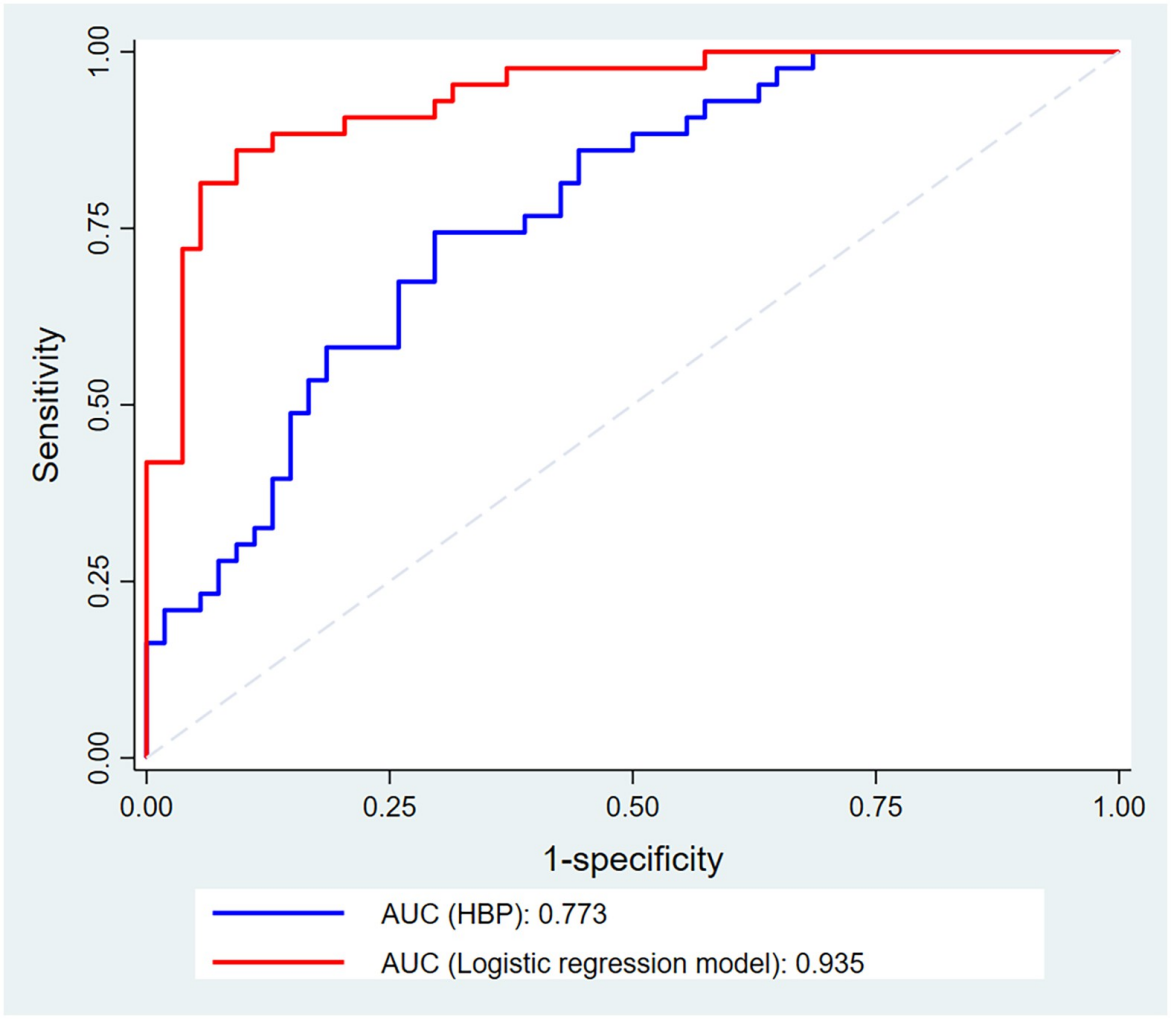
ROC curves for heparin-binding protein and multivariable logistic regression models.

ROC analysis was used to assess the predictive effectiveness of HBP and multivariate logistic regression models. The results showed that the Area Under Curve (AUC) for HBP alone to predict infection in polytrauma was 0.773 (the optimal cut-off value of HBP was 18.08, with a sensitivity of 86.05%, a specificity of 55.56%, and a Yordon index of 0.416 (AUC = 0.773, 95% CI: 0.677~0.852). ROC curves were plotted for the multivariate logistic regression model for comparison, and the results showed that the multivariate logistic regression model had an AUC of 0.935 (95% CI:0.870~0.977), and its predictive efficacy was better than that of HBP alone (Fig 2).

## Discussion

HBP, secreted predominantly by neutrophils, is involved in the septic inflammatory response through antimicrobial action, chemotaxis, and increased vascular permeability. However, the potential value of HBP in polytrauma combined with bacterial infections is uncertain. To clarify the predictive value of HBP for bacterial infections in polytrauma, we utilized the parameter test and univariate logistic regression to analyze the correlation between HBP and infection in polytrauma. The forward stepwise regression was utilized to screen for clinically valuable variables, a multivariate logistic regression model was constructed, and the model was tested. Our study shows that HBP is a potential predictor of bacterial infection in patients with severe polytrauma. Combining HBP, PCT, and neutrophils may improve the prediction of bacterial infection.

This study found that HBP concentrations were higher in the infected group than in the control group, indicating a link between HBP and bacterial infections. Several studies in related fields demonstrated that HBP has antibacterial activity against gram-negative and gram-positive bacteria [16–18]. As shown in the research of B. Lauritzen et al., the antibacterial activity of HBP may be related to its amino acids 20–44 acting as antibacterial domains [19].

The antimicrobial effect of HBP may be related to the involvement in the early immune response to bacterial infection [20, 21]. However, the involvement of HBP in the inflammatory response is not independent. Studies have shown that the increase in IL-26 after infection may promote the release of aggregated HBP from neutrophils [5]. The released HBP binds to glycosaminoglycans on the surface of endothelial cells and triggers endothelial cell contraction and tight junction redistribution, thereby inducing vascular leakage [22]. The involvement of HBP in immune response is further underscored by its role in bacterial phagocytosis. HBP and human neutrophil peptides 1–3 (HNP1–3) enhance macrophage phagocytosis of IgG-opsonized bacteria, acting via β2 integrins [23]. This suggests that HBP not only contributes to the initial immune response but also regulates subsequent phagocytic activity. Moreover, HBP is detected by a TIFA-dependent signalling cascade, linking cytosolic detection of HBP with the common innate immune signalling hub TRAF6, which is essential for signalling downstream of many PRRs [24]. This pathway is specific to HBP, highlighting its unique role in immune sensing and response. HBP is a key player in the pathophysiology of severe bacterial infections, influencing both the initial immune response and subsequent inflammatory processes.

HBP in combination with other indicators of inflammation may have better predictive value in predicting bacterial infections. PCT and CRP, as commonly used clinical indicators for diagnosing bacterial infections, have certain limitations when used individually as compared to their combined use. PCT is detectable 2 hours after bacterial infection and peaks within 6–8 hours [25]. CRP is elevated at 4–6 hours of the inflammatory response and peaks at 36–50 hours [13]. As non-infectious factors can also lead to elevated inflammatory factors, and different inflammatory markers are time-dependent, the combined detection of inflammatory factors is crucial for early warning [26]. For example, an inflammatory complex model combining HBP, PCT, and CRP can more easily and effectively predict the occurrence of acute severe pancreatitis in advance [13]. Therefore, multifactorial analysis models considering the interaction between variables are more conducive to precision medicine. Our findings on the predictive value of HBP combined with other inflammatory indicators agree with those reported by Jin Ma et al., who discovered that adding HBP to other inflammatory markers enhanced prediction accuracy [27].

The role of HBP as an inflammatory factor in infectious diseases has been progressively identified and confirmed [28]. For the first time, we showed the predictive value of HBP for bacterial infections in patients with polytrauma. HBP has good predictive value for bacterial infections in polytrauma patients. As an affordable and immediate test that can be implemented, combining HBP with neutrophils and PCT makes clinical practice more efficient. Therefore, HBP provides a better laboratory basis for early assessment of the condition of trauma patients. However, our study had several limitations. First, as this was a single-centre study, multicentre studies with larger sample sizes are needed to validate HBP’s predictive value further. Second, the condition of patients with polytrauma in emergency medicine is urgent and complex, so we could not classify and analyze them according to the injury site. This study focused on the predictive value of HBP in the early stages of bacterial infection after polytrauma and did not address the changing pattern of HBP over time after infection, which could be studied further. Third, the role of HBP in non-bacterial infections such as viruses, parasites, and fungi needs to be investigated and validated. The next step is to optimize experimental plans and expand experimental samples.

## Conclusions

In patients with severe polytrauma, heparin-binding protein may predict bacterial infection. Combining heparin-binding protein, PCT, and neutrophils may improve bacterial infection prediction. HBP provides a referenceable test for early disease assessment.
